# Identification of Five m6A-Related lncRNA Genes as Prognostic Markers for Endometrial Cancer Based on TCGA Database

**DOI:** 10.1155/2022/2547029

**Published:** 2022-05-04

**Authors:** Li Shan, Ye Lu, Cheng-Cheng Xiang, Xiaoli Zhu, Er-Dong Zuo, Xu Cheng

**Affiliations:** Department of Oncology, Soochow University Affiliated Taicang Hospital (The First People's Hospital of Taicang), Jiangsu 215400, China

## Abstract

**Background:**

Due to difficulties involved in its early diagnosis and adequate prognostication, uterine corpus endometrial carcinoma (UCEC) is one of the most serious threats to human health, with the five-year survival rate being as low as roughly 60%. The discovery of specific biomarkers that serve as prognosticators of UCEC is of great significance. The role of N6-methyladenosine- (m6A-) related long noncoding RNAs (lncRNAs) in the pathogenesis of UCEC remains undefined. In this study, we explored the expression profiles of m6A-related lncRNAs of patients with UCEC and identified novel prognostic markers for UCEC.

**Methods:**

Gene expression and clinical data were extracted from The Cancer Genome Atlas. Coexpression analysis was performed to identify m6A-related lncRNAs, which were entered into univariate Cox regression models for evaluating the prognosis of UCEC. Clusters of UCEC patients and enrichment pathways were identified using consistent data clustering and gene set enrichment analysis (GSEA). A risk score model was established, and Kaplan–Meier analysis was conducted for investigating overall survival (OS) across two patient groups (high risk and low risk). Lastly, the relationship between the risk score and the cell content of 22 types of immune cells, clusters, age, programmed cell death 1 ligand-1 (PD-L1) expression level, immune score, and pathological grade was analyzed.

**Results:**

We identified a total of 2084 lncRNAs associated with m6A, of which 32 lncRNAs were prognostically relevant. Two clusters (clusters 1 and 2) of patients with UCEC were defined; patients in cluster 1 were found to have significantly higher pathological grades and shorter overall survival time compared to those in cluster 2. GSEA showed that “MITOTIC SPINDLE and other pathways” were more enriched in cluster 1. Five major lncRNAs associated with m6A were screened out, and risk score modeling was used for UCEC prognosis prediction. High risk scores were associated with a shorter OS. The risk score was also verified as an independent prognostic indicator for UCEC and was related to immune cell infiltration levels. Finally, we observed a higher pathological grade and greater levels of PD-L1 in the high-risk group than in the low-risk group of patients.

**Conclusions:**

m6A-related lncRNAs play an important role in UCEC progression. The risk-based model constructed from the five key m6A-related lncRNAs was implicated in immune cell infiltration and can potentially be an accurate prognosticator for UCEC.

## 1. Introduction

The incidence of uterine corpus endometrial carcinoma (UCEC), the fourth most common cancer in women, has been increasing in the United States, making it the fifth most common cause of death [1]. Despite rapid advances in medical technology over the years, there has been no notable improvement in the five-year survival rate for UCEC, which was 83.18% in 2015 and 81.81% in 1985 [2]. This lack of improvement is due to early diagnostic difficulties as well as the predominance of high-grade histological tumors [3]. Although the incidence of UCEC is increasing, there are no dependable predictive biomarkers to identify patients with the highest risk of recurrence of the disease, thereby curtailing the application of effective personalized therapy. Thus, it becomes crucial to study the occurrence and development of endometrial cancer at a molecular level. This study focused on screening biomarkers for prognosis prediction of UCEC.

N6-methyladenosine (m6A) is a type of RNA epigenetic modification that can influence tumor development by epigenetic regulation of oncogene and tumor suppressor gene expression during tumorigenesis [4, 5]. The m6A regulators are controlled by regulatory proteins, specifically demethylases (“erasers”), signal transducers (“readers”), and methyltransferases (“writers”) [4]. Historically, m6A has been related to the occurrence and development of cervical, breast, hepatocellular, and endometrial cancers [6–9].

Long noncoding RNAs (lncRNAs) are linear RNAs, often 200 nucleotides long, which regulate the expression of genes at the posttranscriptional or transcriptional level. Aberrant expression of lncRNAs is known to be strongly involved in the development of malignancy [10]. lncRNAs are important in endometrial cancer progression [11]; for example, the lncRNA ASLNC04080 is markedly elevated in UCEC, and its downregulation suppresses cell proliferation and promotes apoptosis by mediating G1 phase arrest [12]. Similarly, H19 was found to be upregulated in endometrial cancer and was correlated with its progression [13]. Elevated HOX antisense intergenic RNA was associated with lymphatic lumen invasion and shortened overall survival in UCEC [14].

Based on concurrent studies, lncRNAs are considered to have a mechanistic function in regulating the modification of m6A and in the development of cancers such as glioma and hepatocellular carcinoma [15, 16]. However, the mechanism underlying the lncRNA dysregulation of tumor cells by m6A is yet to be discovered, with hardly any studies investigating its role in UCEC onset and progression. With the development of bioinformatics, we believe that understanding lncRNAs that are related to m6A may facilitate the discovery of novel biomarkers and therapeutic targets for UCEC [17, 18].

This work investigated m6A-related lncRNAs, which, we hypothesize, are related to the prognosis of endometrial cancer. Using The Cancer Genome Atlas (TCGA), two clusters of endometrial cancer patients were identified via consistent clustering analysis, and their relationship with clinicopathological features was analyzed [19]. We identified five key m6A-related lncRNAs that affect prognosis based on analysis with least absolute shrinkage and selection operator (LASSO) Cox regression [20, 21]. This information was pipelined to develop a model according to risk scores for the prognosis prediction of endometrial cancer. We discovered the critical role of m6A-related lncRNAs in endometrial cancer progression and found that patient prognosis could be predicted accurately using our risk score model.

## 2. Materials and Methods

### 2.1. Case Data

In April 2021, the gene expression data of 552 UCEC cases and 23 healthy samples were obtained from the official TCGA website, of which 542 cases included information on age, pathological classification, and survival time. Based upon previously published literature, 23 m6A-related genes were identified. Separate expression data was obtained from TCGA database for the following genes: eight methyltransferases (*RBM15*, *ZC3H13*, *RBM15B*, *WTAP*, *METTL16*, *METTL3*, *VIRMA*, and *METTL14*), two demethylases (*FTO* and *ALKBH5*), and 13 signal transducers (*YTHDC2*, *YTHDF2*, *RBMX*, *YTHDF1*, *IGF2BP3*, *FMR1*, *LRPRRC*, *HNRNPC*, *YTHDF3*, *IGF2BP1*, *HNRNPA2B1*, *IGF2BP2*, and *YTHDC1*).

### 2.2. Identification of Prognostic m6A-Related lncRNAs and Consistent Construction Analysis

Under Pearson's *R* > 0.5 and *p* < 0.001, the R package “limma” was employed for coexpression analysis to identify m6A-related lncRNAs. The R package “igraph” was used to plot the coexpression network. The m6A-related lncRNAs of prognostic significance were obtained by applying one-way Cox regression analysis (*p* ≤ 0.01). Based on the m6A-related lncRNA expressions, we performed consistent clustering analysis for clustering patients with UCEC. Cluster data was pipelined for Kaplan–Meier (KM) analysis to investigate the differences in OS between clusters. The R plug-ins “pheatmap” and “limma” were applied to analyze the differences between different clusters according to the expression levels of m6A-related lncRNAs, age, pathological grade, and programmed cell death 1 ligand-1 (PD-L1) levels.

### 2.3. Gene Set Enrichment Analysis (GSEA)

Pathway analysis was conducted using the GSEA software (v4.0.3). The false discovery rate (FDR) *q* values < 0.25 and nominal (NOM) *p* values < 0.05 were considered to be of statistical significance.

### 2.4. Construction and Evaluation of Risk Score Models

The patient samples were grouped into the test and training groups in a random manner. For filtering major m6A-related lncRNAs of prognostic significance in the training group, a variant of Cox regression, LASSO regression, was performed. Training group coefficients were determined using the minimum standard method (least 10-fold cross-validation assessment penalty parameter). Based on the m6A-related lncRNAs screened, a risk score model was developed using the following equation:

(1) $$\begin{array}{c} \text{Risk score}=\overset{n}{\underset{i=1}{\sum }}\text{C}\text{oef}i*xi, \end{array}$$

where Coef*i* is the coefficient and *xi* refers to the fragments per kilobase of transcript per million (FPKM) value of a given major m6A-related lncRNA of prognostic significance.

Based on the mean risk scores, UCEC samples were categorized into two risk groups—low and high. Next, survival analysis was done and receiver operating characteristic curve and risk plots were constructed to evaluate the efficiency of the risk score. The risk score was also verified on test groups. Univariate and multivariate Cox regression analyses were employed to assess the impact of the risk score as well as other clinical characteristics on the OS. For patients with varying clinicopathological characteristics, the prognostic capabilities of the risk score were evaluated using the R package “survminer.”

### 2.5. Relationship between the Risk Score and the Immune Cells, Immune Score, Cluster, Age, Pathological Grade, and PD-L1 Levels

The cell content of all 22 kinds of immune cells was calculated using “CIBERSORT” in the R package for each sample. The immune scores of patients with UCEC were estimated using the R packages “estimate” and “limma.” The correlation between the risk score and the immune cells was assessed using the R package “limma.” Further, the correlations between the risk score and the immune score, cluster (identified by consensus clustering analysis), expression of key m6A-related lncRNAs, age, pathological grade, and PD-L1 levels were analyzed with the R packages “pheatmap” and “limma.”

### 2.6. Statistical Analysis

R software v4.0.5 (The R Foundation for Statistical Computing, Vienna, Austria) was employed for data analyses. Unless specified, statistical significance was defined at *p* < 0.05. Cardinality testing was done to evaluate the correlation of clinicopathological characteristics with various groups. The associations between angiogenesis and all factors were explored by univariate and multifactorial Cox analyses. Data were visualized using the R package “ggplot2.”

## 3. Results

### 3.1. Patient Characteristics

Our patient cohort comprised 542 patients with UCEC, with a median age of 64 years (31–90). Among these patients, 98 had UCEC G1, 120 had G2, and 324 had G3 (Table 1). The mean follow-up time was 888 days (range: 0–6, 859 days).

### 3.2. Prognostic m6A-Related lncRNAs in UCEC

**Figure 1:**
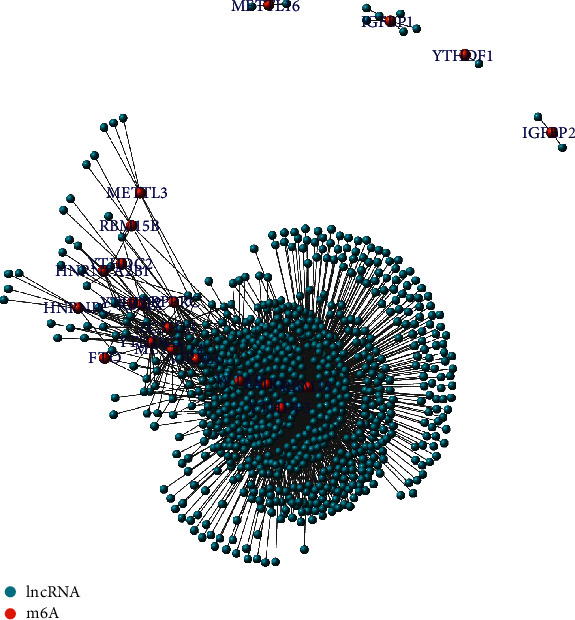
Network of N6-methyladenosine (m6A) as red nodes and 1080 long noncoding RNAs (lncRNAs) as blue nodes.

**Figure 2:**
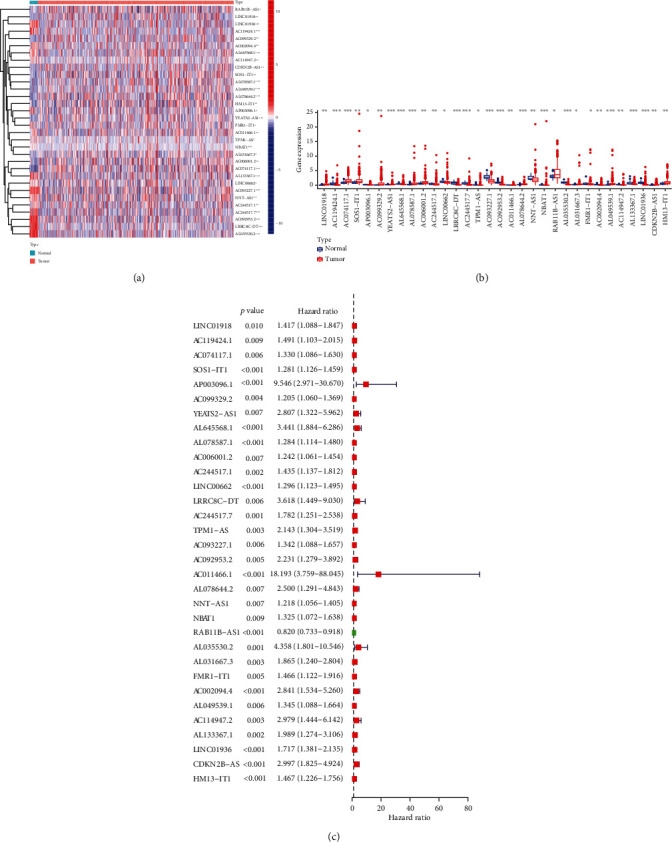
Univariate Cox regression analysis identified 36 prognostic N6-methyladenosine- (m6A-) relevant long noncoding RNAs (lncRNAs). (a) Heatmap of expression of m6A-relevant lncRNAs of prognostic significance. (b) Violin diagram of expression of m6A-relevant lncRNAs of prognostic significance. (c) Univariate regression analysis shows the hazard ratio and forest plot. ^∗^*p* < 0.05; ^∗∗^*p* < 0.01; ^∗∗∗^*p* < 0.01.

A total of 32 m6A-related lncRNAs were found to be prognostically significant from 2084 m6A-related lncRNAs, as seen via coexpression analysis (Figure 1) and pipelining into univariate Cox regression models. The expression of lncRNAs in nontumorous and tumorous tissues and the associated hazard ratios are illustrated in Figure 2.

### 3.3. Patient Clusters Identified by Consistent Clustering Analysis

**Figure 3:**
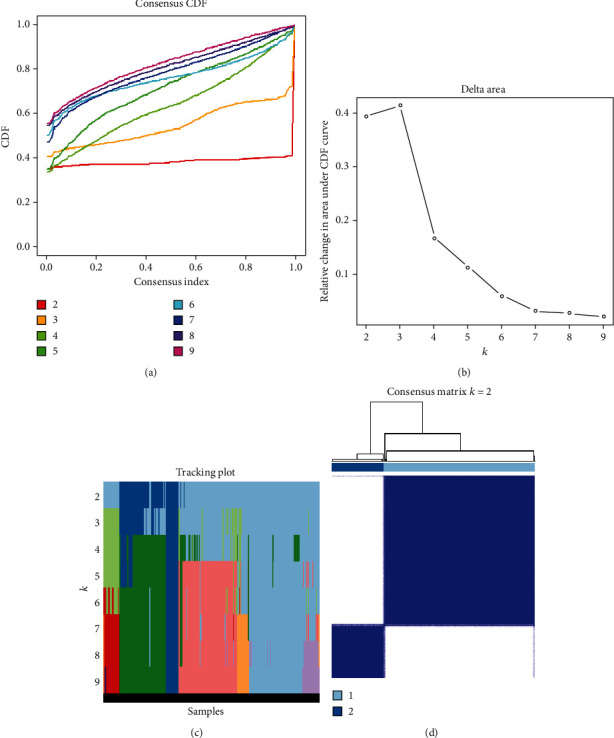
Consensus clustering on the uterine corpus endometrial carcinoma The Cancer Genome Atlas cohort according to the expression of 36 prognostic m6A-relevant lncRNAs. (a) Consensus clustering distribution function (CDF) for *k* = 2-9. (b) Increment in the area under the CDF curve for *k* = 2-9. (c) Tracking plot for *k* = 2-9. (d) Consensus matrix for the optimal value, i.e., *k* = 2.

Figures 3(c), 3(a), and 3(b) show the trace plot of subgroups from *k* = 2-9, the cumulative distribution function (CDF) of consistent clustering from *k* = 2-9, and the relative change in the area under the curve (AUC), respectively. We identified two UCEC clusters (*k* = 2, clusters 1 and 2) according to the association between the maximum increment in AUC and the expression of m6A-related lncRNAs of prognostic significance in subclusters (Figure 3(d)).

### 3.4. Differences in the Expression Levels of m6A-Related lncRNAs with respect to Age, Pathological Grading, and PD-L1 Levels across Clusters

**Figure 4:**
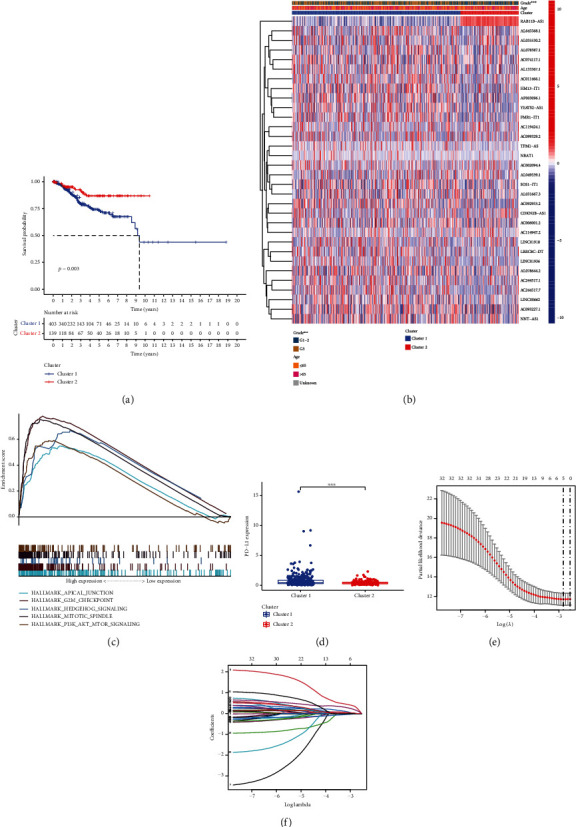
Variation of pathways, patient clinical characteristics, and prognosis among different clusters. (a) Kaplan–Meier overall survival curves of different clusters of uterine corpus endometrial carcinoma (UCEC) patients. (b) Expression of lncRNAs and clinical characteristics in different clusters of UCEC patients. (c) Pathways enriched in cluster 1 in comparison with cluster 2. (d) The expression of programmed cell death 1 ligand-1 (PD-L1) expression among different clusters of patients with UCEC. (e) Least absolute shrinkage and selection operator (LASSO) analysis. (f) LASSO coefficient for nine m6A-relevant long noncoding RNAs. (b) Parameter selection and adjustment in the LASSO model by cross-validation.

KM survival analysis showed that cluster 2 had a remarkably prolonged OS relative to cluster 1 (*p* = 0.003; Figure 4(a)). Furthermore, we investigated the expression of lncRNAs and clinicopathological characteristics in different clusters. As visible in the heatmap, the pathological grading of patients in cluster 1 was considerably higher than that in cluster 2, and various clusters showed different expressions of prognostic lncRNAs (Figure 4(b)).

### 3.5. GSEA

GSEA revealed that G2M CHECKPOINT, PI3K AKT MTOR SIGNALING, and “MITOTIC SPINDLE, MTORC1_SIGNALING, and HEDGEHOG SIGNALING” were more enriched in cluster 1 than in cluster 2 (Table 2, Figure 4(c)).

### 3.6. Development of a Risk Score Model Using Five Key m6A-Related lncRNAs for UCEC Prognosis Prediction

**Figure 5:**
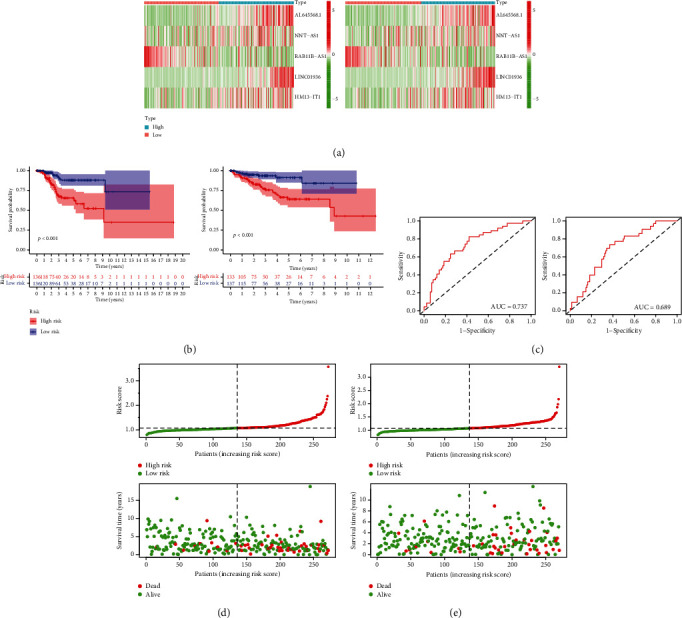
(a) Expression heatmap of major m6A-relevant lncRNAs of prognostic significance in two risk groups (high and low). (b) Patients in the two risk groups (high and low) in the training and test groups by Kaplan–Meier analysis. (c) The ROC curves of patients showing high and low risk scores in the training and test groups (AUC = 0.74 and 0.69, respectively). (d, e) The risk plots and survival of high-risk and low-risk patients in the training and test groups. AUC: area under the curve; ROC: receiver operating characteristic.

**Figure 6:**
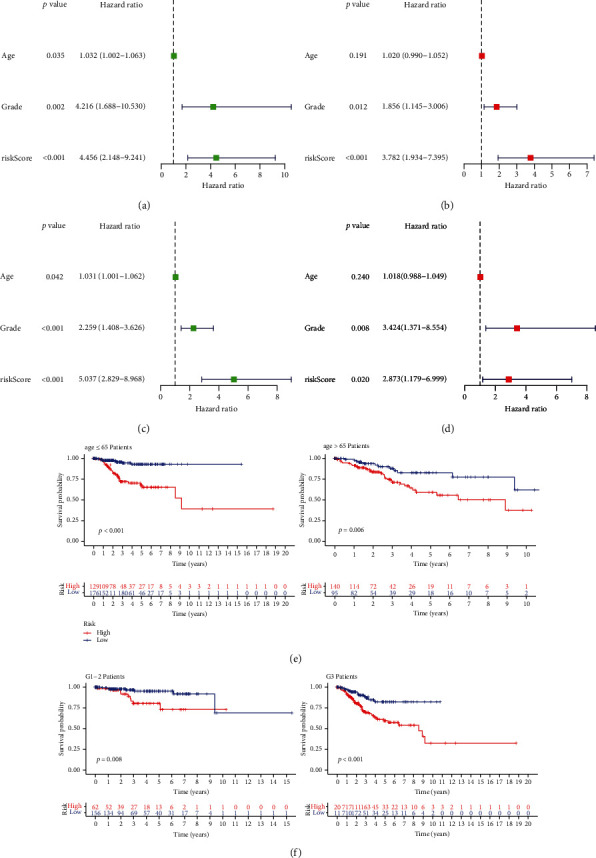
(a, b) Univariate Cox regression analysis of the training group and test group. (c, d) Multivariate Cox regression analysis of the training group and test group shown by forest maps. (e, f) Prognostic significance of the risk score in relation to varied clinicopathologic features: age and grade.

From the training group, five major prognostic m6A-related lncRNAs were screened. For the risk score model development (Figures 4(e) and 4(f), Table 3), coefficients of these lncRNAs and FPKM values were employed. Patients with a high risk score in both the training and test groups tended to have a shorter survival time, as shown by survival analysis data (Figure 5(b)). For 3-year OS, the AUC (Figure 5(c)) was 0.74 and 0.69 for the training and test groups, respectively. Moreover, the risk score could distinctly distinguish between the high- and low-risk groups, as shown by survival state and risk plots (Figures 5(d) and 5(e)). According to univariate and multivariate analyses, the risk scores were independently predictive of survival in both groups (Figures 6(a)–6(d)). Finally, the prognosis was evaluated based on the risk score of patients with varying clinicopathological characteristics: the risk score signature had a high discriminatory value for patient prognosis among different ages and pathological grades (Figures 6(e) and 6(f)).

### 3.7. Relationship between the Risk Score and the Immune Cells, Immune Score, Cluster, Age, Pathological Grade, and Checkpoint Expression Level

**Figure 7:**
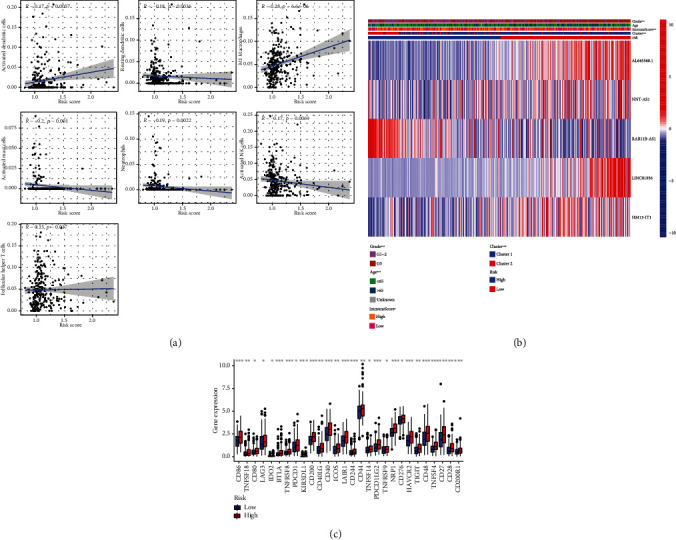
(a) Correlation of the risk scores and immune cells. (b) Relationship between the key long noncoding RNA expressions and the grade, age, immune score, and clusters between different patient risk groups. ^∗^*p* < 0.05; ^∗∗^*p* < 0.01; ^∗∗∗^*p* < 0.01. (c) Expression of the immune checkpoint in different risk score groups.

Risk scores were negatively associated with resting dendritic cells, neutrophils, mast cells, and natural killer (NK) cell activation but positively correlated with dendritic cell activation, M1 macrophages, and follicular helper T cells (Figure 7(a)). Risk scores in cluster 1 patients with a poor prognosis were higher than those in cluster 2 patients with a good prognosis; high-risk patients had profusely higher pathological grading and checkpoint expression levels (Figures 7(b) and 7(c)).

## 4. Discussion

UCEC is a serious threat to human health, and due to the difficulties involved in early diagnosis and estimation of prognosis, the five-year survival for patients with locally advanced malignancy is still about 69%, and for those with distant metastases, it is about 17%, despite therapeutic advances in recent years [22]. Thus, the discovery of specific biomarkers for prognosis prediction of endometrial cancer is of great significance.

Several recent studies have explored biomarkers for UCEC. Jiang et al. created a novel model with nine metabolism-related genes to predict the prognosis of UCEC [23]. Ouyang et al. developed a prognosis prediction model with seven lncRNA genes for UCEC [24].

In this study, the prognostic value of m6A-related lncRNAs was studied in 542 patients with UCEC based on TCGA data. Coexpression analysis filtered 2084 m6A-related lncRNAs, which were then subjected to univariate Cox regression analysis. Here, 32 prognostic lncRNAs were found. Based upon consistent clustering analysis of differential expression of these m6A-related lncRNAs of prognostic significance, two patient clusters were formed. The patients in the 1st cluster exhibited higher pathological grades and worse OS. This suggests that this method can clearly distinguish between UCEC patients with different prognoses and that m6A-related lncRNAs have important functions in UCEC pathogenesis. The results from GSEA showed that “G2M CHECKPOINT, HEDGEHOG SIGNALING, and MITOTIC SPINDLE, PI3K AKT MTOR SIGNALING, MTORC1_SIGNALING” was more enriched in cluster 1 than in cluster 2. G2M CHECKPOINT and MITOTIC SPINDLE are implicated in DNA damage repair and cell cycle regulation, and aberrations in these pathways are often associated with tumor occurrence and progression [25, 26]. PI3K AKT MTOR SIGNALING and HEDGEHOG SIGNALING pathways were verified to be implicated in the occurrence and development of UCEC [27, 28]. The activated MTORC1 pathway has also been found to promote the development of UCEC [29]. However, the specific mechanisms underlying the upregulation of these pathways, especially the relationship between their upregulation and m6A-related lncRNAs, demands further investigation.

Furthermore, we identified five key prognostic m6A-related lncRNAs from the training group using LASSO regression analysis. Among these lncRNAs, NNT-AS1 has been found to be associated with several tumors such as lung, bladder, and prostate cancers [30–32]. Through its natural antisense transcript RAB11B, RAB11B-AS1 inhibits osteosarcoma progression [33]. Wang et al. found that the low expression of RAB11B-AS1 is related to an unfavorable prognosis in UCEC [34]. Similarly, LINC01936 was found to be potentially related to lung adenocarcinoma prognosis [35]. The specific role of AL645568.1 and HM13-IT1 in tumorigenesis, especially UCEC development, needs to be explored further.

In this study, a risk score model was constructed using coefficients and the FPKM value of major m6A-related lncRNAs showing prognostic significance. The model was further validated in the test and training groups. The risk scores were independent and reliable factors for UCEC prognosis, as shown by the results of KM analysis, ROC curves, risk map analysis, and univariate and multivariate Cox regression analyses. The risk score signature was observed to have a high discriminatory value for patient prognosis among different ages and pathological grades. Patients with a high risk score had a higher probability of having a higher pathological grade and shorter OS, and cluster 1 patients with high risk showed a higher risk score than low-risk cluster 2 patients. In conclusion, our results showed that the observed key prognostic m6A-related lncRNAs might have critical functions in the progression of UCEC and, on a larger scale, demonstrated the importance of risk score models and their accuracy in UCEC prognostic prediction.

In our risk score model, patients with a low risk score had higher immune scores than those with a high risk score, indicating that the immune cell content decreased with increasing risk. Macrophages and dendritic cells are important components of the antigen presentation system [36]. The risk scores presented a negative correlation with resting dendritic cells but a positive correlation with activated dendritic cells and M1 macrophages, indicating that elevated risk scores may be associated with disorders of the antigen presentation system. However, the specific mechanism remains to be further confirmed. Mast cells play different roles in different tumors and may promote or inhibit tumor growth [37]. Increased mast cell infiltration is associated with a poor prognosis in lung and colorectal cancers but with a favorable prognosis in breast and prostate cancers [38–40]. Neutrophils also play different roles in different tumors. For example, neutrophils inhibit tumor appreciation and lymphatic metastasis in Epstein-Barr virus-associated gastric carcinoma [41], whereas they promote tumor appreciation and metastasis in colorectal and breast cancers [42, 43]. NK cells are cells with powerful cytolytic functions and play a critical role in host defense against tumors [44]. Our study demonstrated that the risk scores were negatively correlated with neutrophils, activated NK cells, and activated mast cells, suggesting that all three act in an inhibitory manner in UCEC development. Recently, the involvement of follicular helper T cells in cancer development and progression has been increasingly acknowledged. Germinal center follicular helper T cells are a key cell type involved in the formation and maintenance of the germinal center, which enables B cell proliferation and somatic hypermutation and is related to an unfavorable prognosis in gastric and lung cancers [45–47]. Based upon the positive correlation of follicular helper T cells with risk scores demonstrated in this study, this immune cell may be associated with a poor prognosis in UCEC. PD-L1 expression is noticeably elevated in both high-risk groups and high-risk cluster 1 expression. Overexpression of PD-L1 can protect tumor cells from CD8 T cell death; therefore, PD-L1 might be a biomarker for predicting immunotherapy efficiency [48–51]. Therefore, the risk score may be a prognosticator as well as an indicator of immunotherapy efficiency.

There are a few limitations of this study. Primarily, there was relatively little clinical information on patients with UCEC in TCGA database, especially TNM staging, which may lead to potential statistical errors. Second, further experiments are required to assess the efficiency of the prediction model in clinical practice to provide a reliable prognostic prediction for patients with UCEC.

In conclusion, our study is, to our knowledge, the first to demonstrate m6A-related lncRNA expressions in UCEC and their prognostic value. m6A-related lncRNA expression is strongly associated with clinical features and poor survival. Our study, therefore, provides a wealth of evidence and clues to further the study of m6A-related lncRNA's mechanistic role in UCEC.

## Figures and Tables

**Table 1:** Characteristics of patients with renal clear cell carcinoma on The Cancer Genome Atlas.

Clinical characteristic	*N* (542)
Age at diagnosis (years)	64 (31-90)
Grade	
G1	98
G2	120
G3	324

**Table 2:** Different pathways between distinct clusters.

Collection	Name	NES	NOM *p* value	FDR *q* value
Cluster 1 vs. cluster 2	HALLMARK_MITOTIC_SPINDLE	2.504	<0.001	<0.001
	HALLMARK_G2M_CHECKPOINT	2.175	0.002	0.002
	HALLMARK_PI3K_AKT_MTOR_SIGNALING	2.138	0.002	0.003
	HALLMARK_HEDGEHOG_SIGNALING	2.078	<0.001	0.003
	HALLMARK_MTORC1_SIGNALING	2.109	<0.001	0.006

NES; NOM: nominal; FDR: false discovery rate.

**Table 3:** Key prognostic N6-methyladenosine-relevant long noncoding RNAs and their coefficients (Coef).

Name	Coef
AL645568.1	0.347
NNT-AS1	0.001
RAB11B-AS1	-0.020
LINC01936	0.181
HM13-IT1	0.023

## Data Availability

The data used to support the findings of this study are included within the article.
